# 

**Published:** 1916-09-23

**Authors:** 


The Admiralty and the Army Council have now published instructions regarding the issue of the silver badge to officers and others of His Majesty's Naval Forces and the Imperial Military Forces who have served since August 4, 1914, and who, on account of old age or physical infirmity arising from wounds or sickness caused by service, have retired or been discharged from the Navy or Army. A leaflet containing full instructions will be available at all post-offices within a few days, and should be carefully read before making application, so as to ensure that the correct authority is addressed.

### Matrons' and Sisters' Appointments.

Lysaght Auxiliary Hospital, Burnham.—Miss Catherine Blackler has been appointed matron. She was trained at the South Devon and East Cornwall Hospital, Plymouth, where she was later staff nurse and sister, and has since been sister at the Essex and Colchester Hospital, and at two private nursing homes. For the past fifteen months Miss Blackler has been in charge of the military ward at the South Devon and East Cornwall Hospital, Plymouth.

Denbighshire Infirmary.—Miss Mary L. Williams has been appointed matron of this institution, where she was trained. She has been staff nurse at Putney Hospital, theatre sister at Gloucester Royal Infirmary, and sister and assistant matron at Victoria Hospital, Blackpool.

St. Catherine's General and Marine Hospital, Ontario, Canada.—Miss Florence M. Mace has been appointed matron. She was trained at Liverpool Royal Infirmary and at the Ladies' Charity and Lying-in Hospital, Liverpool. She has since been district midwife to the Ladies' Charity and Lying-in Hospital, Birkenhead, and ward sister and theatre sister at Highfield Military Hospital, Liverpool. Miss Mace has also done private nursing.

Abbotts Hospital, Cheltenham.—Miss Bagnall-Oakeley has been appointed lady superintendent. She was trained at Charing Cross Hospital, and has lately been lady superintendent of Hillfield V.A.D. Hospital, Gloucester.

**NOTE.—Particulars of Appointments for insertion in this column will be welcomed.**

### Gallantry at Sea.

### HONOURS FOR NAVAL SURGEONS.

Evidence of the efficiency of the Naval Surgical Service is to be found in the long list of fleet-surgeons mentioned in Sir John Jellicoe's dispatch as having performed distinguished service in the Jutland Bank battle. Acting on Sir John Jellicoe's recommendation, the King has been graciously pleased to give orders for the following appointments to the Distinguished Service Order: Fleet-surgeon J. A. Moon, R.N.; Fleet-surgeon A. Maclean, M.B., R.N.; Fleet-surgeon H. W. Finlayson, M.B., R.N.; Fleet-surgeon B. R. Bickford, R.N.; and Staff-surgeon J. McAlister Holmes, M.B., R.N.

Surgeon-probationer D. G. P. Bell, R.N.V.R., receives the Distinguished Service Cross. Of this officer Captain Walter L. Allen remarks that he devoted great attention to the wounded, and amputated a limb single-handed in the dark.

The following officers are commended for their services in the battle: Fleet-surgeon J. H. Pead, M.B., M.A., R.N.; Fleet-surgeon H. P. Jones, R.N.; Fleet-surgeon A. R. H. Skey, M.B., R.N.; Fleet-surgeon J. R. Muir, M.B., R.N.; Surgeon H. E. R. Stephens, M.B., R.N.; Surgeon (temporary) H. P. Margetts, R.N.; Surgeon-probationer (temporary) G. Blurton, R.N.V.R.; Surgeon-probationer (temporary) N. Macleod, R.N.V.R.; Surgeon-probationer (temporary) C. K. Cullen, R.N.V.R.

Fleet-surgeon R. Hill, C.V.O., principal medical officer of the Fleet Flag-ship and on Admiral Sir John Jellicoe's Staff, has been promoted Deputy Surgeon-General. The Admiral reports that this officer's excellent organisation and services before and after the action were of great assistance, and contributed much to the well-being of the wounded. The following officers have been noted for early promotion: Fleet-surgeons A. Maclean, M.B.; E. A. Penfold, M.B.; A. R. Bankart, C.V.O., M.B.; and C. L. W. Bunton; and Staff-surgeon A. R. Schofield, M.B. Temporary Surgeon R. S. Carey is to be transferred to the permanent list. Surgeon W. J. A. Quine, M.B., R.N.V.R., has also been noted for early promotion. All these officers have a fine record, and Sir John Jellicoe's remarks on the conduct of Fleet-surgeon E. A. Penfold are especially worthy of note. This officer was in the fore medical distributing station when a heavy shell burst just outside, killing and wounding many. Fleet-surgeon Penfold was himself knocked down and bruised and shaken, but personally assisted in the removal of wounded and afterwards tended the wounded with unremitting skill and devotion for fifty hours without rest. His example was invaluable in keeping up the moral of the wounded and of the medical party under very trying conditions.

The President of the French Republic has bestowed the decoration of the Legion of Honour on two Fleet-surgeons—namely, T. Austen, R.N., and J. H. Pead, M.B., R.N.

### WOUNDED ALLIES RELIEF COMMITTEE.

On Thursday, September 14, at noon, the Italian Ambassador received on behalf of the Italian War Office the motor field operating theatre presented by the Wounded Allies' Relief Committee, Sardinia House, Kingsway, to Italy. The presentation took place in the garden of Aldford House, Park Lane, W., the residence of the Hon. Mrs. Frederick Guest (hon. treasurer to the committee, who was present), where the operating theatre was previously on view to the public for several days. With the Italian Ambassador was his wife, the Marchesa Imperiali, and the committee was represented by Sir Lindsey Smith (the hon. secretary), Sir William Collins, and Dr. G. B. Clarke.

**Rates of Subscription:** Twelve months, 6/6; Six months, 4/-; Three months, 2/-, post free to any address in the United Kingdom. Abroad, Twelve months, 8/8; Six months, 5/-; Three months, 2/6, post free. The Hospital "Index" to Volume LIX., October 1915 to March 1916, is now ready, price 1½d. per copy, post free. Address The Manager, "The Hospital," The Hospital Building, 28/29 Southampton Street, Strand, London, W.C.

